# The Predictive Role of Serum Levels of Soluble Cell Adhesion Molecules (sCAMs) in the Therapy of Advanced Breast Cancer—A Single-Centre Study

**DOI:** 10.3390/medicina58020153

**Published:** 2022-01-19

**Authors:** Weronika Bulska-Będkowska, Paulina Czajka-Francuz, Sylwia Jurek-Cisoń, Aleksander J. Owczarek, Tomasz Francuz, Jerzy Chudek

**Affiliations:** 1 Department of Internal Diseases and Oncological Chemotherapy, Faculty of Medical Sciences in Katowice, Medical University of Silesia, 40-027 Katowice, Poland; paulinaczajka@op.pl (P.C.-F.); sylwiacison@o2.pl (S.J.-C.); chj@poczta.fm (J.C.); 2 Health Promotion and Obesity Management Unit, Department of Pathophysiology, Faculty of Medical Sciences in Katowice, Medical University of Silesia, 40-752 Katowice, Poland; aowczarek@paintbox.com.pl; 3 Department of Biochemistry, Faculty of Medical Sciences in Katowice, Medical University of Silesia, 40-752 Katowice, Poland; tfrancuz@sum.edu.pl

**Keywords:** breast cancer, soluble cell adhesion molecules, biomarkers, progression-free survival, overall survival

## Abstract

*Background and Objectives:* Soluble cell adhesion molecules (sCAMs) play a significant role in the metastatic potential of breast cancer (BC). They might block lymphocytes and promote angiogenesis and migration of cancer cells. We assessed the usefulness of sCAMs in the prognosis and monitoring of the progression of advanced BC. *Materials and Methods:* We assessed soluble E-selectin, P-selectin, VCAM-1, ICAM-1, EpCAM, IL-6Ra, TNF-R1, and TNF-R2 in 39 women with aBC. Blood samples were obtained at the beginning of the treatment and after 2 months. *Results:* The median progression-free survival (PFS) was 9 months, and overall survival (OS) was 27 months. The higher levels of sICAM-1 (HR = 2.60, *p* = 0.06) and lower levels of sEpCAM (HR = 2.72, *p* < 0.05) were associated with faster progression of aBC. High levels of sEpCAM through the follow-up period were significantly associated with a lower risk of progression (HR = 0.40, *p* < 0.01). We found the independent predictive value of higher than median sICAM-1 levels for PFS (HR = 2.07, *p* = 0.08) and of sVCAM-1 levels for OS (HR = 2.59, *p* < 0.05). *Conclusions:* Our data support the predictive value of sICAM-1 and sVCAM-1 and suggest that they could become markers for tailoring new therapies in aBC. sEpCAM level could be used as an early indicator of response to the therapy.

## 1. Introduction

Breast cancer (BC) is the most common malignancy and one of the leading causes of cancer death among women worldwide. The global incidence of BC is estimated at over 2 million in 2020 [1]. The incidence rates of BC are increasing by 0.5% per year. Approximately 5–10% of the patients have metastases at the time of diagnosis, and 30–40% of women with early breast cancer (eBC) progress to metastatic disease [2,3]. The 5-year overall survival (OS) rate for patients with IV stage of BC is only 20%, and median OS (mOS) is approximately 3 years [2,4]. The development of numerous therapies has improved outcomes, but they are still below social expectations. BC is a heterogeneous disease with currently distinguished five main subtypes based on the expression of estrogen, progesterone, and HER2 receptors on the cancer cells, which are the primary predictors of treatment efficacy [4]. However, biological mechanisms initiating the metastatic cascade and predictors of this process remain poorly understood. It is expected that a better understanding of this mechanism may help to develop new therapeutic options for the management of BC.

Cell adhesion molecules (CAMs) are a group of proteins that play a significant role in the metastatic potential of BC cells [5]. In physiological conditions, CAMs are responsible for maintaining the continuity of tissues through the interaction of cells between themselves and the extracellular matrix. Adhesins participate in the regulation of cell differentiation, proliferation, migration, and apoptosis [6,7]. Impaired functioning of adhesion molecules at any of the stages might contribute to the loss of normal interaction between cells and allow tumour cells for extravasation and formation of metastases [5].

CAMs are physiologically bound to the cell membrane, and after cleavage, soluble forms can be detected in the circulation (soluble CAM, sCAM) [8]. In cancer patients increased level of sCAM is the result of endothelial dysfunction or their overproduction by tumour cells and immune response to cancer [8,9]. The most recognised and studied CAMs include E-selectin, P-selectin, intracellular adhesion molecule 1 (ICAM-1), vascular cell adhesion molecule 1 (VCAM-1), and epithelial cell adhesion molecule (EpCAM).

E-selectin and P-selectin are adhesive molecules with a glycoprotein structure that consists of intracellular cytoplasmic tail, a transmembrane domain, and an extracellular C-type lectin-like domain, an epidermal grows factor-like domain and homological to complement control protein, which differs between selectins [10]. E-selectin plays an important role in neutrophil adhesion to activated endothelial cells, and P-selectin is involved in the initial recruitment of leukocytes to the site of injury during inflammation [10,11].

ICAM-1 and VCAM-1 are members of the immunoglobulin superfamily, and both mediate the adhesion of leukocytes to vascular endothelium [10]. EpCAM is a transmembrane glycoprotein and plays a role in homotypic cell adhesion in epithelia, thereby holding the cells together [12].

Contrary, the function of the soluble forms of CAMs is poorly understood. It was suggested that the shedding of sCAM might enhance metastasis by binding and blocking cytotoxic lymphocytes [8]. Additionally, sCAMs might promote angiogenesis and migration of BC cells to distant organs [13,14,15,16]. The role of sCAMs in the formation of metastasis was confirmed by numerous studies in which high levels of sCAM-1 were found in the plasma/serum of patients with breast [17], colorectal [18], gastric [19], and lung cancer [20], compared to healthy controls. In addition, higher concentrations of sCAMs were also observed in women with BC in comparison to patients with benign breast tumours [21,22,23,24] and patients with distant metastases than in eBC [23].

Therefore, the main goal of the study was to assess the usefulness of the above-mentioned sCAMs in the prediction and monitoring of the response to the therapy of advanced breast cancer (aBC).

## 2. Materials and Methods

### 2.1. Study Group

The study involved 48 unselected Caucasian women with aBC treated in the Department of Internal Diseases and Oncological Chemotherapy, the Medical University of Silesia in Katowice, from July 2014 to September 2019. An inclusion criterion was histologically confirmed aBC (with metastases to distant organs revealed in imaging techniques: computed tomography, magnetic resonance imaging, skeletal scintigraphy, sonography). The exclusion criteria were as follow: a history of autoimmune disease (*n* = 3), venous thrombosis (*n* = 2), infections (*n* = 3), and hemolysis in the first blood specimens (*n* = 2). One of the patients had more than one exclusion criteria. Finally, 39 women were included in the analysis.

The first peripheral blood samples were obtained from 34 women before initiation of therapy (*n* = 23–chemotherapy, *n* = 4–targeted therapy, *n* = 7–hormone therapy with aromatase inhibitors (*n* = 2), tamoxifen (*n* = 3), fulvestrant (*n* = 2)), 4 women shortly after the start of therapy (*n* = 2–chemotherapy, *n* = 1–targeted therapy, *n* = 1–hormone therapy with an aromatase inhibitor), and 1 woman without treatment, qualified for watchful waiting. The second blood sample collection was obtained after 2 months from the first assessment (before the subsequent cycle among treated with chemotherapy). All patients had undergone physical examination and assessment of laboratory parameters before each treatment cycle or visit. Computed tomography or ultrasonography was performed generally every 3 months to assess the treatment efficacy, and the best responses were documented according to the Response Evaluation Criteria in Solid Tumours version 1.1 (RECIST ver 1.1) [25]. The collected data included disease characteristics, the best response to treatment, comorbidities, time to progression, and death.

The study was approved by the Bioethics Committee of the Medical University of Silesia in Katowice (KNW/0022/KB1/2/15, KNW/0022/KB1/2/I/15/16, KNW0022/KB1/2/III/15/16/18/19). Written informed consent was obtained for each patient.

### 2.2. Biochemical Measurements

Five milliliters of peripheral blood was collected in BD Vacutainer Tubes. Obtained blood samples were immediately centrifuged at 3000 rpm for 10 min to obtain serum, then transferred to tubes and immediately stored in the liquid nitrogen.

Serum levels of soluble forms of E-selectin, P-selectin, VCAM-1, ICAM-1, EpCAM, Interleukin-6 Receptor subunit alpha (IL6Ra), Tumor Necrosis Factor Receptor 1 (TNF-R1), Tumor Necrosis Factor Receptor 2 (TNF-R2) were measured via multiplex technique (R&D Systems, Inc., Minneapolis, MN, USA), following the manufacturer’s instruction. Bead fluorescence readings were taken using the Bio-Plex 200 System (Bio-Rad, Hercules, CA, USA) using high PMT (High RP1) setting and analysed with Bio-Plex Manager version 6.1.0.727 (Bio-Rad, Hercules, CA, USA).

### 2.3. Data Analysis

The diagnosis of diabetes was based on fasting serum glucose above 125 mg/dL on two separate tests [26] or medical history and antidiabetic drugs. Patients were considered to have hypertension if they had a mean systolic blood pressure (SBP) ≥140 mmHg and/or diastolic blood pressure (DBP) ≥90 mmHg or used antihypertensive medications [27].

The body mass index (BMI) was calculated as the weight (kg) divided by the square of the height (meters).

Rapid disease progression (RDP) was defined as progression occurring within 6 months from the start of the study.

### 2.4. Statistical Analysis

Statistical analyses were performed using STATISTICA 13.0 PL (TIBCO Software Inc., Palo Alto, CA, USA), StataSE 13.0 (StataCorp LP, College Station, TX, USA), and the R software (R Core Team (2013), R Foundation for Statistical Computing, Vienna, Austria, http://www.R-project.org/ accessed on 17 November 2021). Statistical significance was set at a *p*-value below 0.05. All tests were two-tailed. Imputations were not performed for missing data. Nominal and ordinal data were expressed as percentages. Interval data were expressed as the mean value ± standard deviation in the case of normal distribution. In the case of data with skewed or non-normal distribution, they were expressed as the median, with lower (Q_1_) and upper (Q_3_) quartiles. The distribution of variables was evaluated by the Anderson–Darling test and the quantile-quantile (Q–Q) plot. Homogeneity of variances was assessed by the Levene test. Nominal and ordinal data were compared with the χ^2^ test. Comparisons between groups for interval data and also for longitudinal data were conducted with mixed models (with either raw variables or after logarithmic transformation in the case of non-normal data distribution). The post hoc tests were performed with one-way variance analysis (ANOVA), with the Benjamini–Hochberg correction for multiple testing. Overall survival, as well as progression-free survival, were analysed with the Cox proportional regression models and shown with hazard ratios (HR), corresponding confidence intervals (±95% CI), and *p*-values. We used either the baseline values divided below and above the median (Model 1), standardised values (Model 2), or the time-depended values. The proportionality assumption was tested based on the Schoenfeld residuals (R function *cox.zph*). Multiple-collinearity was checked based on the correlation matrix of coefficients of the survival model. Additionally, PFS was performed with Kaplan–Meyer curves, stratified by median values, with the log-rank test to compare survival curves. The associations between variables were assessed with the Spearman ranks correlation coefficient.

## 3. Result

### 3.1. Study Group Characteristics

The study group consisted of 39 women with aBC aged between 39 and 85 years (average age 60 ± 11 years), and significant comorbidity including hypertension (66.7%), type 2 diabetes (28.2%), and obesity (15.4%)—Table 1.

A greater percentage of patients were diagnosed with hormone-receptor-positive/HER2 negative (HR+/HER2−) breast cancer (66.7%), with metachronous metastases (64.1%) and multiple organs metastases (76.9%). The most common sites of metastases were bones (53.8%) and lungs/pleura (51.3%). Seventeen of 26 patients (65.4%) with HR+/HER− aBC started chemotherapy after progression on hormone therapy.

Anticoagulant prophylaxis and bisphosphonates were prescribed in 35.9% and 30.8% of patients, respectively.

During the follow-up period, the disease progression occurred in 31 (79.5%), and 28 (71.8%) died due to metastatic breast cancer. The rapid disease progression was observed in 17 (43.6%) patients. The median time of progression-free survival (PFS) was 9 months (quartiles: 4–34), and overall survival (OS) was 27 months (quartiles: 14–44).

### 3.2. sCAMs Concentrations and Relationships between Them

The median serum soluble adhesion molecules (sCAMs) concentrations at baseline and after 2 months of follow-up are shown in Table 2. Compared with baseline levels, serum sVCAM-1 and sICAM-1 concentrations after 2 months significantly increased, while the sEpCAM level decreased. Other assessed parameters remained unchanged (Table 2).

**Figure 1:**
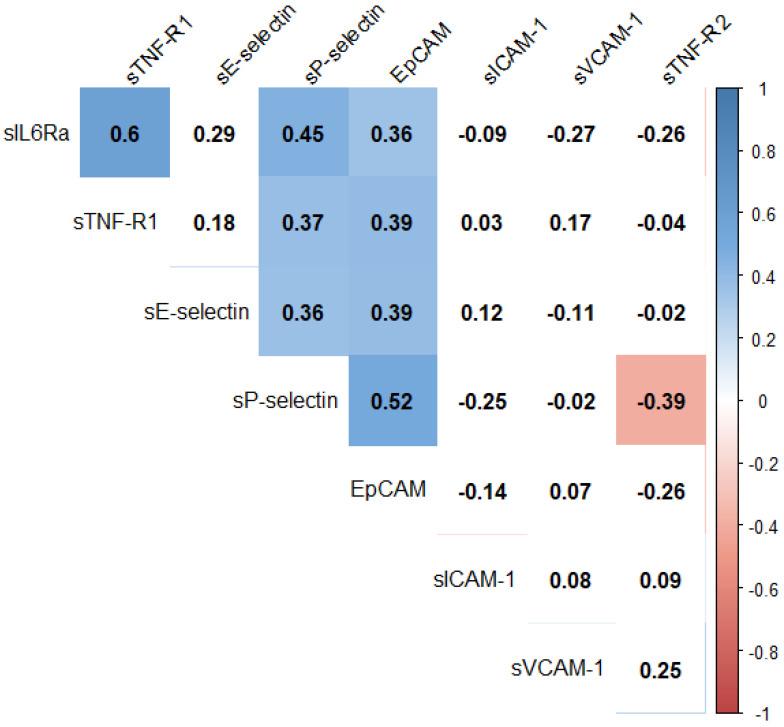
Spearman ranks correlation coefficients between initial values of assessed parameters. The colour represents the direction and strength of the correlation coefficient. A white background indicates no statistical significance.

Significant moderate positive correlations at baseline were found between baseline sP-Selectin and sEpCAM, sE-Selectin as well as sIL-6Ra levels (ρ = 0.52, *p* < 0.01; ρ = 0.36, *p* < 0.05; ρ = 0.45, *p* < 0.01, respectively), while moderate negative one with sTNF-R2 (ρ = −0.39, *p* < 0.05). In addition, from moderate positive correlations between sEpCAM and sE-Selectin, sIL-R6a and sTNF-R1 levels were found (ρ = 0.39, *p* < 0.05; ρ = 0.36, *p* < 0.05; ρ = 0.39, *p* < 0.05, respectively; Figure 1).

In the case of relative changes in the initial value, the moderate positive correlations between sVCAM-1 and sTNF-R1 as well as sTNF-R2 were found (ρ = 0.36, *p* < 0.05; ρ = 0.37, *p* < 0.05, respectively) and between sEpCAM and sP-selectin as well as sIL-6Ra (ρ = 0.37, *p* < 0.05; ρ = 0.32, *p* < 0.05, respectively).

### 3.3. Impact of sCAMS and Clinicopathological Characteristics on Progression-Free Survival

The univariable analysis showed that occurrence of liver metastasis, serum sICAM-1, sVCAM-1, and sTNF-R2 levels above-median were associated with a greater risk of progression. Contrary the HER2 overexpression combined with targeted treatment (trastuzumab ± pertuzumab) declined the risk. Other factors were insignificant (Table 3).

**Figure 2:**
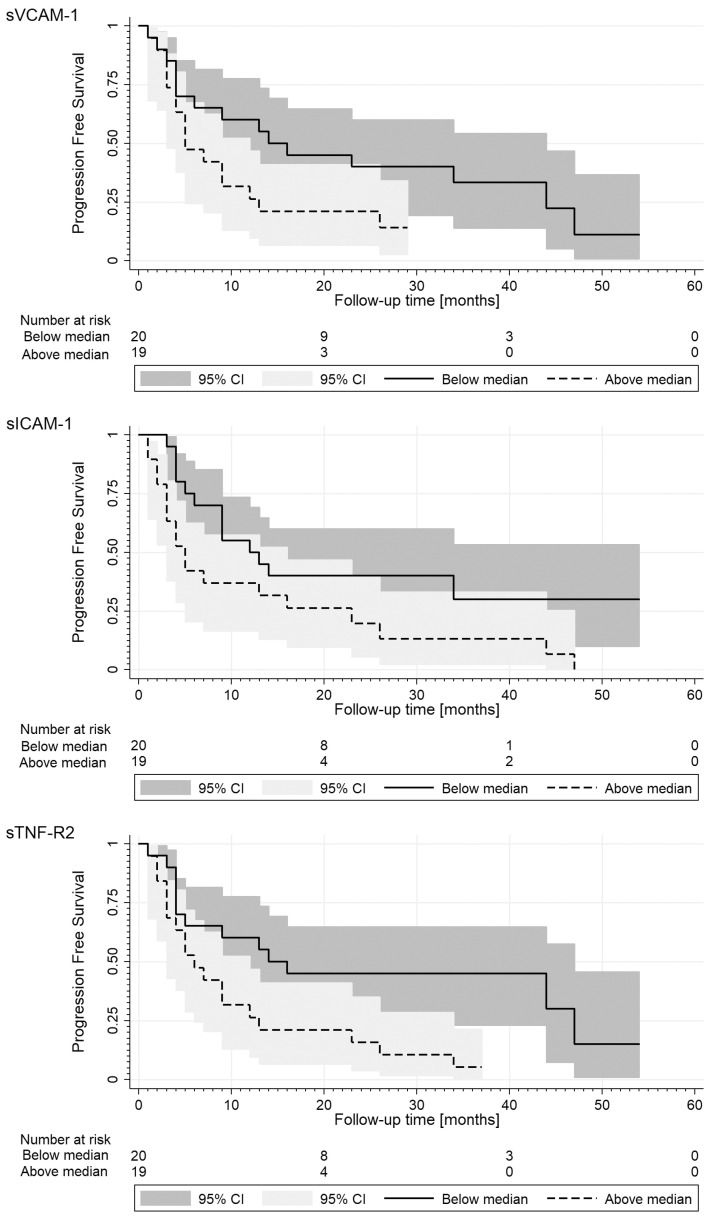
Kaplan–Meier estimates of progression-free survival (PFS) stratified for the median values of sVCAM-1, sICAM-1, and sTNF-R2 levels. Median values of PFS for higher and lower levels are as follows: 14 (4–44) vs. 5 (5–13); 12 (5-) vs. 5 (3–23); 14 (4–47) vs. 6 (3–13) months. The log-rank test *p*-value of survival curves comparison were as follows: 0.07, <0.05 and <0.05, respectively.

In parallel, the log-rank analysis of Kaplan–Meier survival curves performed for the significant biomarkers mentioned above showed that patients with levels above the median of sICAM-1 (>388.5 ng/mL), sVCAM-1 (>624.6 ng/mL), and sTNF-R2 (>1.95 ng/mL) had significantly shorter PFS than patients with lower levels (Figure 2).

Cox proportional hazards models for PFS were used with independent factors selected based on the univariable analysis (Table 3). The multivariable models included: liver metastasis, anty-HER2 targeted treatment, sTNF-R2 levels, and either sICAM-1 levels according to the median (model 1) or its standardised concentrations (model 2). In the first model, only higher levels of sTNF-R2 (*p* < 0.05) and sICAM-1 (*p* = 0.08) increased more than twice the risk of progression. On the contrary, in the second one, the occurrence of liver metastasis maintained the predictive value (*p* = 0.08). Moreover, the time-dependent Cox model showed that high levels of sEpCAM through the follow-up period were significantly associated with a lower risk of progression (HR = 0.40; 95% CI: 0.21–0.77; *p* < 0.01).

### 3.4. Impact of sCAMS and Clinicopathological Characteristics on Overall Survival

The univariable analysis showed that occurrence of liver and multiple organ metastasis was associated with a greater risk of progression, brain metastasis, higher grading, and sVCAM-1 levels above-median were of borderline significance. On the contrary, the HER2 overexpression combined with targeted treatment (trastuzumab ± pertuzumab) declined the risk. Other factors were insignificant (Table 3). In parallel, the log-rank analysis of Kaplan–Meier survival curves performed for sVCAM-1 showed that patients with levels above the median had a shorter OS (median values: 23 (9–33) vs. 32 (23–47) months; *p* = 0.08).

Cox proportional hazards models for OS were used based on factors selected in the univariable analysis (Table 3). The multivariable model included: occurrence of liver and multiple organ metastasis, grading and anty-HER2 targeted treatment, and sVCAM-1 levels according to the median. Both the occurrence of multiple organ metastasis and sVCAM-1 levels maintain predictive value for OS (Table 3). No significant factors were identified by the time-dependent Cox model.

### 3.5. Sensitivity Analyses

There were 17 (43.6%) fast progressors in the study group, and they had more than 3-times higher risk of death (HR = 3.47, 95% CI: 1.59–7.61; *p* < 0.01). The higher levels of sICAM-1 (HR = 2.60, 95% CI: 0.96–7.07; *p* = 0.06) and lower levels of of sEpCAM (HR = 2.72, 95% CI: 0.99–7.51; *p* < 0.05) were associated with fast progression. The mixed model’s analysis with repeated measurements showed that there is a significant effect of slow/fast progression and time for sEpCAM levels (*p* < 0.001), with significant interaction. The post hoc adjusted analysis revealed that sEpCAM levels diminished through the follow-up period only in slow progressors (1029 (757–1442) vs. 575 (495–909); *p* < 0.05). Moreover, at baseline, they had higher levels than fast progressors (607 (443–744), *p* < 0.01). No difference was observed after a 2 month follow-up period.

The sub-analysis of luminal HER2-negative aBC (*n* = 26) showed that although the higher levels of sICAM-1 and sTNF-R2 above the median, as well as sICAM-1 standardised levels, lost a statistical significance, yet kept a predictive direction for PFS (HR: 1.30, 1.80, 1.09, respectively). Similarly, in case of OS the sVCAM-1 levels kept its predictive direction (HR = 2.19; 95% CI: 0.84–5.70; *p* = 0.11).

## 4. Discussion

In the present study, we analysed serum levels of soluble cell adhesion molecules and their potential prognostic value for PFS and OS in a single-centre cohort of women with aBC. We showed that assessment of sICAM-1 is useful for the prediction of PFS and sVCAM-1 for the prediction of OS, besides routinely assessed receptor status and antigen Ki67. The sensitivity analysis restricted to the luminal HER2 negative aBC—the most common subtype—showed a similar pattern; however, the findings were not statistically significant, probably due to the small size of the study cohort.

Previously published data suggested an association between sCAM concentrations and survival, but the results were inconsistent. Contrary to our results, several researchers demonstrated the prognostic value of sICAM-1 and sE-selectin for OS [28,29,30]. While, similarly to our results, Bewick M. et al. [28] demonstrated worse survival and shorter PFS in patients with higher levels of sVCAM-1 in the univariable analysis. In addition, they showed that serum level of sICAM-1 was an independent predictor for both PFS and OS. Notwithstanding, we did not confirm the effect of sICAM-1 on OS. The differences in the obtained results between studies might be due to the small size of study groups and tumour heterogeneity. Of note, an association was found between sCAM and clinicopathological parameters such as grade, stage of the disease [31] but not hormone receptor status of BC [28,29]. Our study cohort was not big enough to assess eventual differences in the concentration of sICAM-1 and sVCAM-1 between biological subtypes of aBC.

We also observed that sICAM-1 and sVCAM-1 levels changed during the treatment. The cause of the increase in the concentration of sCAM after 2-month of cancer therapy (mostly chemotherapy) could be damage of endothelium by cytotoxic chemotherapeutics. Similar results were observed in the group of patients with early BC receiving adjuvant chemotherapy. Mills et al. [32] showed elevated sICAM-1 levels at the start of cycle 4 as compared with pretreatment values. In the current study, chemotherapy regimens used in aBC were based on anthracycline, cyclophosphamide, and taxanes. It could be expected that the mechanisms of endothelial damage are different depending on the type of agent. Doxorubicin causes endothelial cell retraction with exposure of subendothelial matrix in vascular endothelial cell monolayers and induces endothelial cell apoptosis [33,34]. Moreover, paclitaxel and cyclophosphamide are cytotoxic for endothelial cells [34,35]. In turn, the treatment with selective estrogen receptor modulators (SERMs), as shown by Tesarova et al. [36], is followed by a decrease in the concentrations of sVCAM-1, sICAM-1, and sP-selectin 3 months and 1 year after the initiation of the treatment. This means that sCAM originated from tumour cells diminished as the result of hormone therapy. In turn, cytotoxic agents lead to an increase in the concentration of sCAM as a result of its sheading from damaged endothelium. That excludes their role in predicting the response to therapy in patients on chemotherapy. The decrease in sCAM would probably be observed during the treatment with new generation drugs such as CDK 4/6, PI3K, and PARP inhibitors. The hypothesis requires confirmation in further researches.

These cell adhesion molecules are located primarily on the surface of the endothelium and cells of the immune system. CAMs expression is induced by cytokines such as TNF-α [37]. However, we did not observe the correlation between sTNF-R2 and the above-mentioned sCAMs, but sTNF-R2 was an independent predictive factor for PFS in aBC. In previous studies, it was shown that sTNF-R2 was associated with postmenopausal breast cancer risk [38].

To the best of our knowledge, this study was the first that investigated the association between levels of sEpCAM and survival in patients with aBC. Karabulut et al. [17] conducted a similar study but enrolled patients with all stages of BC, including 37% with disseminated disease. They observed a lack of a prognostic value of sEpCAM on PFS and OS. Furthermore, the current study is the first one in the literature that investigated the changes in the serum level of sEpCAM during cancer therapy. We showed that baseline levels of sEpCAM had no prognostic value, but a decrease in sEpCAM concentration during the treatment was observed in patients with slow progression, thus probably with a better response to therapy. In addition, only patients with high baseline sEpCAM level benefited from the treatment, mostly chemotherapy. Thus far, studies on the predictive value of the soluble form of EpCAM have not yet been performed. Most of the studies were devoted to the expression of EpCAM in tissues of epithelial-derived neoplasms [39,40,41]. Survival was estimated to be worse in patients with higher expression EpCAM in breast cancer tissue [39], especially in the luminal B HER2 positive and basal-like breast cancer [42,43], and ovarian [44], prostate [45], and gall bladder cancers [46]. In physiological conditions, this molecule plays a role in homotypic cell adhesion and is also involved in the cell differentiation and cell cycle by its capacity to upregulate cyclins A and E [47]. However, the origin, significance, and function of its soluble form are unclear. Possible sources of elevated serum levels of sCAM include tumour cells [48]. The correlation between the stage and grade of breast cancer and overexpression of EpCAM in the tumour tissue was observed [41,43]. However, Karabulut et al. [17] did not find any difference in sEpCAM serum levels both in women with advanced BC and early BC but revealed its higher levels in cancer patients compared to healthy controls.

In summary, we confirmed the prognostic value of sICAM-1 and sVCAM-1 in patients with aBC. In addition, we evaluated the changes in the serum levels of sCAM, and similar to the first, we found the association between time of progression and changes in sEpCAM levels. However, we observed an increase in sICAM-1 and sVCAM-1 during the treatment, largely based on CTH. Thus, the usefulness of these sCAM as a marker of response to chemotherapy is limited.

This study has some limitations, mostly related to the size of our single-centre cohort. In consistence with the cancer statistics, most of our patients had luminal cancer subtypes, and therefore we restricted sensitivity analysis to this subtype. We cannot exclude that sCAM profile and its prognostic value might be different for non-luminal subtypes of aBC. Only a multicenter study can investigate sCAMs profile in patients with all breast cancer subtypes and assess its prognostic value. This problem might also be solved by access to samples collected and stored during previously performed studies. Thus, performing assessments in the stored samples may also overcome the limitations of our study.

In addition, the study was conducted when CDK4/6 inhibitors were not available outside clinical trials. Most patients with HR+/HER2− cancer received chemotherapy after failure of adjuvant hormone therapy. Currently, CDK4/6 inhibitors are the main therapeutic option in the treatment of luminal HER2 negative aBC as the first-line or second-line [49]. Further analysis of clinical trials would be required to determine whether sCAM could be used to optimise the sequence of therapy with CDK4/6 inhibitors.

## 5. Conclusions

(1) Our data support the predictive value of sICAM-1 and sVCAM-1 and suggest that they could become markers for tailoring new therapies in aBC;

(2) Serum sEpCAM levels might have a diagnostic role in patients with aBC and be used as an indicator of response to CTH. Additionally, it could be helpful to identify patients with luminal breast cancer who will have the benefit from chemotherapy.

However, we have stressed that this study has a preliminary nature. These hypotheses require confirmation in a much larger multicenter clinical trial.

## Figures and Tables

**Table 1:** Characteristics of the study group. Data shown as mean values ± SD or medians (Q_1_–Q_3_).

Age	60 ± 11
BMI (kg/m^2^)	26.4 ± 4.2
BMI ≥ 30, *n* (%)	6 (15.4)
Metastasis:	
synchronous, *n* (%)	14 (35.9)
metachronous, *n* (%)	25 (64.1)
Metastasis to:	
one organ, *n* (%)	9 (23.1)
multiple organs, *n* (%)	30 (76.9)
Bone metastasis, *n* (%)	21 (53.8)
Liver metastasis, *n* (%)	14 (35.9)
Brain metastasis, *n* (%)	4 (10.3)
Lung/pleural metastasis, *n* (%)	20 (51.3)
Soft tissue metastasis/thoracic infiltration, *n* (%)	11 (28.2)
Metastasis to the mediastinal and retroperitoneal lymph nodes, *n* (%)	17 (43.6)
Grading	
1, *n* (%)	0
2, *n* (%)	26 (66.7)
3, *n* (%)	9 (23.1)
no data *n*	4
Ki67, %	34 ± 27
EgR, *n* (%)	28 (71.8)
PgR *n* (%)	19 (48.7)
HER2, *n* (%)	10 (25.6)
Biological subtypes of breast cancer:	
HR+/HER2−, *n* (%)	26 (66.7)
HR−/HER2−, *n* (%)	4 (10.3)
HR−/HER2+, *n* (%)	5 (12.8)
HR+/HER2+, *n* (%)	4 (10.3)
Hypertension, *n* (%)	26 (66.7)
Diabates mellitus t.2, *n* (%)	11 (28.2)
Bisphosphonates, *n* (%)	12 (30.8)
Insulin therapy, *n* (%)	2 (5.1)
Antithrombotic prophylaxis, *n* (%)	14 (35.9)
Follow up, months	23 (9–32)
The best first response to treatment:	
PD, *n* (%)	15 (38.5)
SD, *n* (%)	16 (41.0)
PR, *n* (%)	8 (20.5)
PFS, months	9 (4–34)
Observation time in the living patients, months	32 (26–39)
Deaths, *n* (%)	28 (71.8)
OS, months	27 (14–44)

**Table 2:** Serum concentrations of sCAMs and other assessed parameters at baseline and after 2 months of follow-up.

	Baseline	After 2-Month Follow-Up	Δ (%)	*p*
sE-selectin, ng/mL	21.0 (14.3–23.0)	21.6 (16.4–25.9)	6.3 ± 45.3	0.39
sP-selectin, ng/mL	38.1 (31.9–55.8)	41.1 (39.4–48.2)	11.0 ± 46.2	0.17
sVCAM-1, ng/mL	624.6 (460.3–923.9)	814.2 (684.6–1072.0)	32.6 ± 54.5	<0.05
sICAM-1, ng/mL	388.5 (314.9–484.1)	464.8 (379.0–534.6)	23.4 ± 52.5	<0.05
EpCAM, ng/mL	0.76 (0.57–1.37)	0.57 (0.51–0.68)	−13.3 ± 42.9	<0.05
sIL6Ra, ng/mL	3.38 (2.33–6.67)	3.11 (2.57–3.85)	−1.7 ± 46.8	0.11
sTNF-R1, ng/mL	0.84 (0.61–1.11)	0.93 (0.69–1.13)	22.6 ± 87.6	0.54
sTNF-R2, ng/mL	1.95 (0.98–2.50)	1.92 (1.40–2.12)	27.7 ± 91.4	0.57

Data are shown as mean values ± SD or medians (Q_1_–Q_3_). Δ—relative percentage change of the initial value.

**Table 3:** Univariable and multivariable analysis of progression-free survival (PFS) and overall survival (OS) in aBC. Model 1 included statistically significant clinical variables and raw baseline factors values, while model 2 had significant clinical variables and baseline standardised values.

HR (±95% CI)	Univariable		Multivariable		
	PFS	OS	PFS		OS
			Model 1	Model 2	Model 1
Age [yrs]	0.98 (0.94–1.01)	0.98 (0.95–1.02)	-	-	-
Diabetes mellitus	1.64 (0.74–3.64)	1.19 (0.52–2.73)	-	-	-
Grade 3 vs. 2	1.52 (0.66–3.51)	2.27 (0.94–5.51) ^$^	-	-	1.10 (0.4–3.09)
Multiple organ metastasis	1.78 (0.72–4.40)	2.70 (1.01–7.25) *	-	-	3.73 (1.24–11.21) *
Luminal HER2 negative	1.27 (0.58–2.77)	2.06 (0.86–4.94)	-	-	-
Bone metastasis	1.02 (0.50–2.07)	1.40 (0.65–3.04)	-	-	-
Liver metastasis	2.04 (0.98–4.28) ^$^	3.06 (1.38–6.82) ^#^	1.32 (0.53–3.31)	2.01 (0.93–4.37) ^$^	1.89 (0.70–5.15)
Brain metastasis	1.88 (0.64–5.49)	2.86 (0.93–8.82) ^$^	-	-	-
Transtuzumab ± peruzumab therapy (HER2 positive)	0.36 (0.14–0.96) *	0.20 (0.06–0.70) *	0.81 (0.31–2.08)	0.98 (0.42–2.27)	1.56 (0.55–4.39)
HT vs. CTH	0.58 (0.22–1.53)	0.74 (0.28–1.96)	-	-	-
Baseline					
sE-selectin > 21.0 ng/mL	1.66 (0.79–3.47)	1.55 (0.71–3.36)	-	-	-
sP-selectin > 38.1 ng/mL	0.57 (0.27–1.17)	0.61 (0.28–1.35)	-	-	-
sVCAM-1 > 624.6 ng/mL	1.96 (0.92–4.18) ^$^	1.96 (0.91–4.21) ^$^	-	-	2.59 (1.15–5.82) *
sICAM-1 > 388.5 ng/mL	2.07 (1.01–4.24) *	1.42 (0.66–3.08)	2.07 (0.90–4.72) ^$^	-	-
EpCAM > 0.76 ng/mL	0.70 (0.33–1.45)	1.23 (0.55–2.74)	-	-	-
sIL6Ra > 3.38 ng/mL	0.74 (0.36–1.55)	0.71 (0.32–1.58)	-	-	-
sTNF-R1 > 0.84 ng/mL	0.83 (0.41–1.70)	1.39 (0.65–2.99)	-	-	-
sTNF-R2 > 1.95 ng/mL	2.48 (1.16–5.30) *	1.88 (0.87–4.06)	2.48 (1.16–5.30) *	-	-
sE-selectin per 1-SD	1.17 (0.85–1.63)	1.30 (0.91–1.85)	-	-	-
sP-selectin per 1-SD	0.76 (0.48–1.20)	0.87 (0.55–1.37)	-	-	-
sVCAM-1 per 1-SD	1.21 (0.86–1.70)	1.24 (0.88–1.75)	-	-	-
sICAM-1 per 1-SD	1.60 (1.08–2.38) *	1.18 (0.82–1.68)	-	0.90 (0.62–1.30)	-
EpCAM per 1-SD	0.88 (0.58–1.31)	1.28 (0.84–1.95)	-	-	-
sIL6Ra per 1-SD	0.73 (0.48–1.12)	0.71 (0.44–1.15)	-	-	-
sTNF-R1 per 1-SD	0.78 (0.49–1.23)	0.92 (0.56–1.53)	-	-	-
sTNF-R2 per 1-SD	1.29 (0.96–1.74)	1.18 (0.88–1.59)	-	-	-

^$^ *p* < 0.1, * *p* < 0.05, ^#^ *p* < 0.01.

## Data Availability

The data presented in this study are available on request from the corresponding author. The data are not publicly available due to subject privace restrictions.
